# Underlying Mechanisms of Brain Aging and Neurodegenerative Diseases as Potential Targets for Preventive or Therapeutic Strategies Using Phytochemicals

**DOI:** 10.3390/nu15153456

**Published:** 2023-08-04

**Authors:** Hamid Mostafavi Abdolmaleky, Jin-Rong Zhou

**Affiliations:** Nutrition/Metabolism Laboratory, Department of Surgery, Beth Israel Deaconess Medical Center, Harvard Medical School, Boston, MA 02215, USA; sabdolma@bidmc.harvard.edu

**Keywords:** aging, neurodegeneration, microglia, neuroinflammation, phytochemicals, molecular target, gut microbiota, neural stem cell, metabolism

## Abstract

During aging, several tissues and biological systems undergo a progressive decline in function, leading to age-associated diseases such as neurodegenerative, inflammatory, metabolic, and cardiovascular diseases and cancer. In this review, we focus on the molecular underpinning of senescence and neurodegeneration related to age-associated brain diseases, in particular, Alzheimer’s and Parkinson’s diseases, along with introducing nutrients or phytochemicals that modulate age-associated molecular dysfunctions, potentially offering preventive or therapeutic benefits. Based on current knowledge, the dysregulation of microglia genes and neuroinflammation, telomere attrition, neuronal stem cell degradation, vascular system dysfunction, reactive oxygen species, loss of chromosome X inactivation in females, and gut microbiome dysbiosis have been seen to play pivotal roles in neurodegeneration in an interactive manner. There are several phytochemicals (e.g., curcumin, EGCG, fucoidan, galangin, astin C, apigenin, resveratrol, phytic acid, acacetin, daucosterol, silibinin, sulforaphane, withaferin A, and betulinic acid) that modulate the dysfunction of one or several key genes (e.g., TREM2, C3, C3aR1, TNFA, NF-kb, TGFB1&2, SIRT1&6, HMGB1, and STING) affected in the aged brain. Although phytochemicals have shown promise in slowing down the progression of age-related brain diseases, more studies to identify their efficacy, alone or in combinations, in preclinical systems can help to design novel nutritional strategies for the management of neurodegenerative diseases in humans.

## 1. Introduction

Aging is a normal process in the life of any species, but some individuals experience early or premature aging and thus, advanced age-associated diseases impacting the quality of their life accompanied by enormous economic and social burdens. Therefore, it would be rational to mitigate aging processes, not only to support healthy aging but also to hamper age-associated diseases. During aging, different functional systems are affected in an interactive manner [1]. These include the central nervous system (CNS), cardiovascular system, immune system, and muscles, among others. In reality, as we get older, in addition to an insidious neurodegenerative and cognitive decrement, the functionality of the cardiovascular system is diminished by cholesterol and calcium precipitation and inflammation of the endothelial cells [2]. Similarly, the functionality of the immune system is affected, making the elderly vulnerable to cancer, infectious diseases, and activation of retrotransposons residing in the genome, inducing inflammation [3]. Along with these maladies, the musculoskeletal system is also prone to progressive weakening, causing movement problems that could intensify the aforementioned complications and increase the risk of all types of dementia, including Alzheimer’s disease [4]. 

In humans, Alzheimer’s disease (AD) and Parkinson’s disease (PD) are the most common and important age-associated CNS diseases. Almost 15% of individuals exhibit symptoms of AD around the age of 70 years, while at the age of 90, it is nearly 40%, and over the age of 105, almost 65% of individuals are affected [5]. Although the prevalence of PD (almost 1% of the general population) is less than AD, it has an earlier age of onset, usually around 60 years [6], and, similar to AD, it is regarded as a progressive and chronic neurodegenerative and likely neuroinflammatory disease. In AD, the accumulation of misfolded β-amyloid peptide (Aβ) and tau-protein-induced neuropathology is linked to reactive neuroinflammation and secondary widespread neurodegeneration [7]. In PD, misfolded a-synuclein aggregates are responsible for the neuroinflammation of dopaminergic cells of the substantia nigra, while the misfolded a-synuclein may also spread to other brain cells, including cortical regions, leading to insidious cognitive deterioration [8]. 

**Figure 1:**
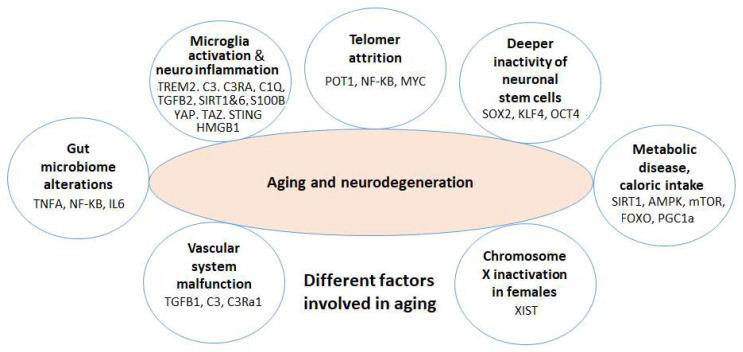
Different interacting internal and external factors are linked to brain aging and neurodegenerative diseases by influencing the function of key genes which could be targeted by various nutrients or phytochemicals.

Regarding the treatment of these debilitating brain diseases, while there have been a number of pharmacological remedies commonly employed in clinical settings in the last few decades, the main challenge is that these drugs have limited efficacy, and, in general, they do not target the underlying mechanisms of disease pathogenesis. However, recent research advancements in the era of genetics and epigenetics addressing the underlying mechanisms of age-related mental diseases, along with many scattered findings exhibiting the effects of nutritional or bioactive compounds/compositions on brain aging, have provided new opportunities to combine these dispersed findings and propose new strategies for the mitigation of or treatment of neurocognitive deterioration during aging. Here, following an updated overview of the molecular and cellular components as well as key dysregulated genes involved in brain aging, as depicted in Figure 1 (which will be addressed in detail in the following sections), we provide an inclusive review related to the different types of dietary ingredients/nutrients and phytochemicals which may help to prevent/postpone or treat these diseases along with a discussion about their mechanisms of action. 

## 2. Methods

For this study, we initially conducted a literature review in PubMed (NIH, National Library of Medicine) and the Web of Science to gather data on the fundamental mechanisms underlying brain aging, neurodegeneration, Alzheimer’s disease, and Parkinson’s disease. We focused on examining original research articles and comprehensive reviews, along with their references, to obtain in-depth information. A list of the most significant factors, supported by multiple studies, and the affected genes (illustrated in Figure 1) was compiled for each condition after a review of almost 2500 articles in PubMed using each keyword of gene expression, epigenetic, or microbiome plus brain aging (~570 articles), neurodegeneration (~600 articles), Alzheimer’s disease (~830 articles), or Parkinson’s disease (~470 articles). This list was continuously updated until April 2023, and additional research articles related to their potential roles in neurodegenerative diseases were searched in the Web of Science as well. Subsequently, we conducted further literature searches using the name of each affected gene as the primary keyword, combined with a single word from the following categories: phytochemical, diet, nutrition, microbiome, microbiota, and microbes in PubMed and the Web of Science. The purpose was to investigate whether the activity of the affected gene is influenced by any of these secondary factors. We found more than 30 original articles which address the desirable effects on each condition as described in the following sections. While our primary focus was on collecting the data of original research articles, which consisted of almost 25% of those >2000 articles mentioned above, we also examined the references of relevant reviews to identify additional original articles that may have been missed in our initial search. Any relevant articles found were added to our collection as appropriate. Supplementary Table S1 exhibits the number of articles (original research articles and reviews combined) studied for this work in PubMed. 

## 3. Key Factors in Brain Aging and Neurodegeneration 

### 3.1. Neuroinflammation and Microglia Dysregulated Genes in AD and the Effects of Phytochemicals

The role of glia activation and other immune cell dysfunction is well described in AD [9]. Single-cell RNA sequencing revealed AD-associated brain transcriptome alterations in specific types of microglia [10]. Peripheral blood single-cell RNA sequencing also uncovered enhanced immune cell signatures and reduced B cell-related molecular biomarkers in AD [11,12]. Additionally, in the search for the identification of the underlying mechanisms of immune cell dysfunction, large-scale whole genome DNA methylation analyses of post-mortem brains and blood samples identified that DNA methylation alterations associated with AD are involved in the dysregulation of the immune system [13]. Moreover, while there is evidence for an accelerated epigenetic aging in AD [14] and by using an “epigenetic clock”, one can reliably estimate cellular/tissue aging [15], more recent studies revealed that the loss of epigenetic information is among one of the key components of events contributing to cellular aging [16,17]. In other studies focusing on important microglia innate immune receptor genes, an increase in the DNA methylation of TREM2 in the superior temporal gyrus was linked to AD pathogenesis [18]. Nevertheless, another study reported that the increased DNA methylation of TREM2 associated with a >3-fold increased expression in the hippocampus was due to the enrichment in 5-hydroxymethycytosine (5-hmC) at the TREM2 gene body [19] where, in general, 5-hmC increases the associated gene expression. There is also a report on the increased TREM2 expression in the blood cells of patients with AD associated with the DNA hypomethylation of several CpGs in its intron 1 [20]. However, there is also evidence that the increased TREM2 expression in the hippocampus might be a secondary protective reaction. In fact, the overexpression of TREM2 using adeno-associated virus vector gene delivery increased the number of Iba-1/Arg-1-positive microglia, suppressed neuroinflammation and microglial activation, and improved cognition in high fat-fed mice exhibiting glucose intolerance as well as learning and memory problems [21]. 

Several studies indicated that phytochemicals could attenuate neuroinflammation and improve cognitive performance involving TREM2 in mice models of AD. For instance, in APP/PSEN1 transgenic AD model mice, it was shown that cognitive dysfunction and hippocampal neuroinflammatory responses are decreased by Bilberry anthocyanins, and the microglia phagocytosis of beta-amyloid protein plaques is improved by upregulation of the TREM2/TYROBP/CD33 signaling pathway [22]. In a more recent study, Cyanidin-3-O-glucoside (C3G), a phytochemical found in fruits and vegetables, could also reduce neuroinflammation and reactive oxygen species (ROS), and enhance microglial Aβ42 phagocytosis through the upregulation of TREM2 in a mouse model of AD [23]. 

**Figure 2:**
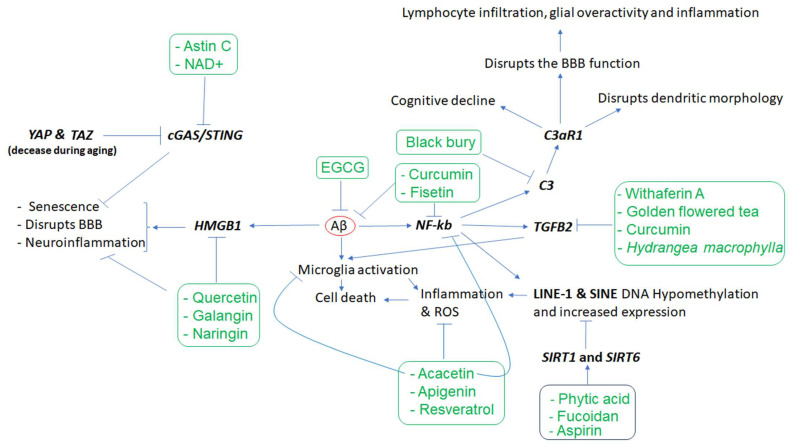
The cascade of events resulting from amyloid-beta (Aβ) accumulation in the brain and the phytochemicals that may mitigate the effects of these events. Aβ appears to play a central role in inducing inflammation, which is mediated by the overactivity of HMGB1, NKB, C3, C3AR, and TGFB2, as well as LINE-1 and SINE transposons, among others. However, several phytochemicals (inside rectangles) have the potential to target the affected genes, reduce inflammation, and mitigate the other cellular dysfunctions that contribute to neuronal death. In the figure, arrows indicate stimulation, while the T-shape marks denote inhibition. Genes are presented in bold.

Considering microglia’s contribution to AD pathogenesis, it is not surprising that other key elements of the immune system, such as complements C1q, C3, and C4 [24,25] along with TGFB2 [26] which regulate synapse pruning [27], are affected in AD. For example, as shown in Figure 2, the Aβ protein activates astroglia NF-κb and releases the complement C3 which acts on neuronal complement C3a receptors (C3aR) and disrupts the dendritic morphology and network functions in AD patients [28]. Other studies reported that complement C3 and C3aR1 are increased by aging, resulting in inflammation and increased permeability of the vascular structure (mediated by Ca++) impacting the blood–brain barrier (BBB) integrity associated with lymphocyte infiltration, and greater microglial activity [29]. Furthermore, as the expression of complement C3 and C3aR1 in the human AD brain are correlated with cognitive decline, the inactivation of C3aR attenuates tau pathology, neuroinflammation, and neurodegeneration in mice models of AD [30].

Epigenetic analyses also uncovered the epigenetic dysregulation of several complement genes, such as C3 [31] and ITGB2/C3R [32], as well as several other proinflammatory genes in the post-mortem brain samples of patients with AD [33]. For instance, the DNA hypomethylation of C3 associated with its increased expression was shown in the post-mortem brains of patients with AD [31]. The DNA methylation alteration of other genes mainly expressed by astrocytes and glial cells (e.g., S100B, the S100 calcium-binding protein B) is also linked to AD pathogenesis [34]. Therefore, the epigenetic regulation of these genes using several nutritional components that act on promoter DNA methylation (e.g., folic acid, vitamin B12, and choline) could be promising remedies to rebalance the activity of affected genes. In fact, while folate and vitamin B12 deficiencies, which result in hyperhomocysteinaemia and dysfunctional DNA methylation machinery, are linked to dementia and other mental diseases, their potential roles in the prevention of AD are highly warranted [35,36].

Notably, aging is associated with DNA hypomethylation and reactivation of transposon elements such as LINE1 and SINE which induce inflammation. Transposons are viral DNA that have been incorporated into the genome of different species and compromise almost 50% of the human genome. Epigenetic mechanisms, including DNA methylation, are responsible for suppressing their activity throughout the life of any animal. It has been shown that SIRT6 is one of the key genes in transposon inactivation, by inducing DNA methylation, and its upregulation suppresses transposon reactivation, leading to longer life in *Drosophila melanogaster* [37]. In humans, its overactive allele is associated with longer life, i.e., becoming a centenarian [38]. Fucoidan, a phytochemical of Atlantic brown algae increases SIRT6 expression by nearly 14-fold [39], increases the lifespan and is neuroprotective in *D. melanogaster* [40,41], and improves LPS-induced cognitive impairment in mice [42]; thus, it is a promising anti-aging phytochemical. 

Beside SIRT6, SIRT1 expression is downregulated by internal brain factors/toxins such as Aβ, accelerating neuronal cell senescence which can be attenuated by exogenous SIRT1 expression as well as aspirin (salicylic acid, a plant hormone) that upregulates SIRT1 expression [43]. Phytic acid was also shown to diminish the dysfunction of these players in cell culture experiments and the aged mice brain [44]. 

In addition, it has been shown that the active compounds of black chokeberry (*Aronia melanocapa* L.) decrease the expression of several inflammatory factors, including ITGB2/C3R in neuronal cells [45]. As ITGB2 is upregulated in the blood mononuclear cells of AD patients, 1α,25(OH)2-vitamin D3 (1,25D3) could promote Aβ phagocytosis through the macrophages of AD patients and decrease inflammation in vitro [46].

Regarding TGFβ2, while its overexpression is reported in the neurons of patients with AD [26], in vitro studies uncovered that its expression is induced by toxic Aβs both in glial and neuronal cells. Interestingly, the increased TGFβ2 protein in the brain binds to the extracellular domain of Aβ precursor protein and activates a neuronal cell death pathway in AD, and the degree of TGFβ2-induced cell death is greater in cells that express a familial AD-related mutation in APP versus those that express the wild-type APP [26,47]. Altogether, these data suggest the potential roles of TGFβ2 dysregulation in the pathogenesis of AD; thus, it is a legitimate target for therapeutic interventions using phytochemicals to suppress neuroinflammation. In this line, there is evidence that the expression of TGFβ2 can be downregulated by certain phytochemicals in other diseases, suggesting their potential use in AD. For example, withaferin A, a steroidal lactone derived from the *Solanaceae* plant family (including eggplants, tomatoes, potatoes, and bell and chili peppers), was shown to inhibit TGFβ2 (and TGFβ1) expression in chronic kidney disease [48]. Active fractions of golden-flowered tea were also shown to decrease the expression of TGFβ2 as well as TGFβ1 and TGFβ3 both in vitro and in vivo [49]. An extract of *Hydrangea macrophylla* could also reduce TGFβ2 synthesis in hair follicles [50]. In neuronal tissue, curcumin exhibits anti-inflammatory effects and suppresses reactive gliosis in animal models of spinal cord injury through decreasing TGFβ1 and TGFβ2, along with proinflammatory cytokines such as TNF-α, IL-1β, and NF-κb [51]. Therefore, more studies on the potential penetration of these phytochemicals into brain tissue can open a path for their application in AD and other neurodegenerative diseases.

High Mobility Group Box 1 (HMGB1), another gene linked to microglia function, is also involved in AD pathogenesis [52]. As HMGB1 is a known marker of neuroinflammation, and the serum level of HMGB1 is higher in AD [53], it may disrupt BBB functions [54]. Tau oligomer also induces HMGB1 release, promoting cellular senescence [55]. Several phytochemicals are known to suppress HMGB1. For instance, quercetin inhibits HMGB1 and reduces cellular reactive oxygen species (ROS) and the apoptotic responses of liver cells involved in the mitochondrial pathway [56]. Naringin was seen to inhibit HMGB1 expression and suppress the expression of proinflammatory cytokines in a mouse model of COVID-19 [57]. Galangin also was shown to suppress the HMGB1/TLR4 bond, mitigating astrocytic activation and neuroinflammation in rat brains [58]. 

Although there is evidence that HMGB1 expression is regulated by DNA methylation in metabolic disease [59,60], there are no such studies in neurodegenerative diseases. However, a number of studies have shown that HMGB1 expression is regulated by HDAC4&5 and miR-129 in brain cells [61,62]. As miR-129 is known to regulate neuronal migration in mice brains [63] and choline (abundant in spinach) upregulates miR-129-5p expression in neural progenitor cells both in vitro and in vivo [64], there are several other phytochemicals and drugs (e.g., gallic acid, sulforaphane, and valproate) which stimulate or inhibit HDAC4 or HDAC5, thus opening new windows for more studies in the treatment of AD and other neurodegenerative diseases.

In addition to the above-mentioned important genes, YAP and TAZ genes (members of the superfamily of ATP-binding cassette transporters), whose activities decline during aging, are other key elements of cellular senescence or aging. As YAP/TAZ, through the suppression of cGAS/STING, preserve the nuclear envelope integrity involving innate immunity and prevent aging, the inhibition of STING (stimulator of interferon genes or TMEM173) prevents tissue aging and senescence-associated inflammation [65]. Likewise, the PQBP1-cGAS-STING pathway is involved in Tau-induced microglia activation, leading to brain inflammation [66]. It has been shown that supplements containing NAD^+^ (and its precursors, abundant in mushrooms, avocados, and cucumbers) could reduce the expression of proinflammatory cytokines and mitigate microglia and astrocyte activation, through decreasing cGAS-STING activity, which was shown to attenuate neuroinflammation and cell senescence in a mouse model of AD [67]. cGAS-STING signaling could be also inhibited by Astin C (a cyclopeptide extracted from the plant of *Aster tataricus*), which has been shown to decrease the innate inflammatory responses triggered by cytosolic DNAs both in vitro and in mice [68]. 

Since STING has been shown to detect cytosolic nucleic acids (e.g., from the death of cells, damaged mitochondria, intrusive bacteria, viruses, and reactivated transposons) and signals to activate the innate immune system, in addition to its involvement in autophagy, it appears to have dual functions [69]. In fact, while STING is involved in the microglia-induced inflammation in reaction to DNA released from the damaged and dysfunctional mitochondria in AD [70], infectious diseases such as chronic hepatitis B decrease the expression of STING due to its promoter DNA hypermethylation [71]. The infection of monocyte-derived macrophages by the highly pathogenic porcine reproductive and respiratory syndrome virus (HP-PRRSV) also decreases the expression of STING [72]. However, in these situations, phytochemicals such as rutin, α-tocopherol, and ascorbic acid could increase its expression but, at the same time, could reduce the expression of TNFα and TGFβ in the affected cells [72].

Lastly, as inflammation and ROS are the most important consequences of the dysfunction of the aforementioned key players, leading to neurodegeneration, importantly, apigenin (a compound mostly found in parsley and celery) was shown to be neuroprotective via decreasing inflammation and ROS in iPSC-derived neurons from patients with AD [73]. Resveratrol from grapes, berries, and peanuts has also demonstrated anti-inflammatory and anti-aging effects [74]. Additionally, epigallocatechin gallate (EGCG), a tea phytochemical, reduces Aβ (1–40), the amyloid precursor protein (APP), and neuronal apoptosis, and activates TrkA signaling (the receptor for BDNF) in the APP/PS1 mice model of AD associated with cognitive improvement [75]. Similarly, curcumin inhibits Aβ plaque formation, decreases the hyperphosphorylated tau, and increases their elimination, in addition to decreasing microglial activity and inhibiting acetylcholinesterase and ROS in several studies [76]. Moreover, acacetin derived from *Robinia pseudoacacia* also has anti-inflammatory activity and protects dopamine neurons against MPTP neurotoxicity both in vitro and in mouse models of MPTP-induced PD [77]. Acacetin was seen to suppress microglial activation and neuroinflammation in an LPS-induced mouse model of neuroinflammation and reduce proinflammatory cytokines (such as TNF-α and IL-1β) and inhibit NF-κb activation in cell culture experiments [78]. Fisetin, a polyphenolic compound found in many fruits (e.g., apples, strawberries, and cucumbers) and vegetables was shown to influence the function of several pathways involving MAPK, PI3K/Akt, Nrf2, NF-κb, and protein kinase C and may also reduce the ROS, neuro-inflammation, and neurotoxicity mitigating neurodegenerative diseases [79]. 

Table 1 and Figure 2 show a summary of findings related to the effects of different phytochemicals with potential application in neurodegenerative diseases on specific genes. However, there are many other phytochemicals that exhibit neuroprotective effects via other mechanisms, but their gene-specific mechanism of action is not well defined. For example, experimental evidence indicates that quercetin exhibits neuroprotective effects in PD through the downregulation of α-synuclein protein aggregation in PD [80] and inhibits the fibril formation of Aβ proteins in AD [81]. Similarly, naringenin could alleviate the neurotoxic effects of Aβ in vitro as it has been associated with the downregulation of the expression of APP and BACE, reducing amyloidogenesis, and decreasing the level of phosphorylated tau [82]. Several other lines of evidence also support the beneficial effects of narigenin in AD and PD [83] as well as the neuroprotective effects of *Bacopa monnieri* extract, with potential therapeutic applications in AD [84]. 

### 3.2. Brain Neuronal Stem Cells (NSCs) in Aging and the Effects of Phytochemicals 

Aging is associated with a decrease in the total NSC population in the hippocampus (from >20,000 in each square millimeter at age 3 months to almost 7000 at the age of 12 months in mice). In addition, the quiescent NSCs (qNSCs), which upon activation enter a primed quiescent state from the dormant state and generate neurons and glia, enter a deeper quiescence state due to aging [85]. Thus, the recovery potential of the aging brain is compromised. 

It has been shown that NSC proliferation is increased by homolocarpum seed oil (a rich source of α-linolenic acid and β-sitosterol), thus indicating it is a potential candidate to counteract brain aging [86]. Likewise, as summarized in Table 2, several other phytochemicals such as daucosterol, a component of walnut meat also boost NSC proliferation mediated by increases in IGF expression and AKT phosphorylation in cell culture experiments [87]. *Alyssum homolocarpum* seed extract has also been seen to increase NSC proliferation in mice brains [88]. Furthermore, Kuwanon V isolated from the root of a mulberry tree (*Morus bombycis*) was shown to increase neurogenesis (from rat NSCs) and cell survival, mediated by the reduction in extracellular signal-regulated kinase 1/2 phosphorylation, increase in p21 expression, Notch/Hairy downregulation, and the upregulation of the microRNAs miR-9, miR-29a, and miR-181a [89]. Silibinin, a polyphenolic flavonoid from *Silybum marianum*, also increases NSC proliferation in mice through BDNF/TrkB signaling transduction [90]. Similarly, a resveratrol pretreatment could increase NSC survival and proliferation and decrease the apoptosis associated with the upregulation of Nrf2, HO-1, and NQO1 protein expression as shown following an oxygen–glucose deprivation/reoxygenation challenge in vitro [91]. Additionally, curcumin was shown to inhibit the bisphenol A-mediated reduction in NSC proliferation and neuronal differentiation by activation of Wnt/β-catenin signaling in the mouse hippocampus [92]. However, in cell culture experiments, curcumin was seen to inhibit NSC differentiation and increase cell survival mediated by decreases in Atg7 and p62 expression, the markers of autophagy [93]. Likewise, the neuroprotective effects of capsaicin and resveratrol against Glu-induced toxicity were shown in the cerebral cortical neurons of mouse fetuses [94]. 

In more recent studies, it has been shown that while key genes encoding basic transcription factors for stemness are active during embryogenesis, they are silenced in later life. However, concurrent increases in the expression of SOX2, OCT4, and KLF4 have been associated with recovering the lost epigenetic information in aged cells and rejuvenation of several tissues, including neuronal cells in mice eyes [95]. Therefore, the induction of the expression of these genes has been shown to be a promising therapeutic approach for the treatment of age-related diseases via recovering epigenetic memory [17,95]. Meanwhile, several phytochemicals are also known to potentiate the expression of these genes. For example, di-(2-ethylhexyl) phthalate (DEHP), a *C. vulgure* phytochemical, increases SOX2 expression in the hippocampal NSC of mice in vitro and is associated with an increased cell growth rate [96]. Furthermore, sulforaphane (a phytochemical compound of broccoli), withaferin A (a steroidal lactone from a medicinal plant), and betulinic acid (a phytochemical from several tree bark extracts) could increase the expression of KLF4 in vitro [97,98,99]. Although many phytochemicals reduce the expression of OCT4, a short-term low-dose ethanol treatment (1 week, 1–5 mM, equivalent to a blood concentration of ~0.0048–0.024%) increases OCT4 expression several folds in vitro [100]. Therefore, an appropriate combination of these phytochemicals with low-dose ethanol may help to increase the expression of these key transcription factors posited to be useful in tissue rejuvenation or regeneration. 

**Table 2:** Phytochemicals that modulate NSC proliferation in neurodegenerative diseases.

Phytochemicals or Nutrients	In Vitro and/or Animal Models	Effects	Mechanisms of Action	References
*Alyssum Homolocarpum* seed oil	Embryonic NSC (eNSC)	- Increases eNCS viability and proliferation	- Increases the expression of notch1, hes-1, and Ki-67	[86]
Daucosterol (walnut meat)	NSC	- Boosts NSC proliferation	- Increases IGF expression and AKT phosphorylation	[87]
*Alyssum homolocarpum (Brassicaceae*) seed extract	Mice brain	- Increases NSC proliferation	Not investigated	[88]
Kuwanon V (from mulberry tree (*Morus bombycis*) root)	Rat NSC	- Increases neurogenesis and cell survival	- Increases p21 expression - Downregulates Notch/Hairy - Upregulates miR-9, miR-29a, and miR-181a	[89]
Silibinin (from *Silybum marianum*)	Mouse	- Increases NSCs proliferation	- Works through BDNF/TrkB signaling transduction	[90]
Resveratrol	NSC	- Increases NSC survival and proliferation	- Upregulates Nrf2, HO-1, and NQO1 protein expression	[91]
Curcumin	Mice hippocampus	- Increases NSC proliferation	- Activates Wnt/β-catenin signaling	[92]
Di-(2-ethylhexyl) phthalate (*C. vulgure*)	NSC of mice hippocampus	- Increases NSC growth rate	- Increases SOX2 expression	[96]
Sulforaphane (broccoli)	In vitro	Unknown	- Increases KLF4 expression	[97]
Withaferin A (a medicinal plant)			- Increases KLF4 expression	[98]
Betulinic acid (bark of trees)			- Increases KLF4 expression	[99]
Short-term low-dose ethanol	In vitro	Unknown	- Increases OCT4	[100]

### 3.3. Telomere Attrition and Aging and the Effects of Phytochemicals 

**Figure 3:**
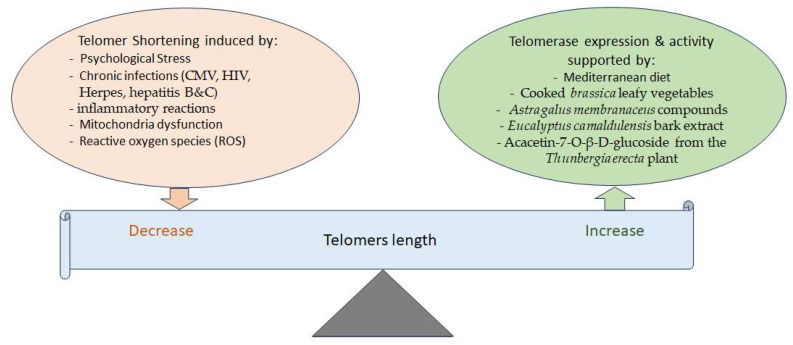
Different factors are involved in balancing telomere length. Telomerase activity helps maintain telomere length, while various factors can decrease telomere length. On the other hand, several phytochemicals and the Mediterranean diet have been shown to increase telomerase expression or activity, thereby potentially increasing or maintaining telomere length.

The shortening of telomere length, which usually occurs during cell replication, is another determinant of aging. It is known that psychological stress, chronic infections, mitochondria dysfunction, and ROS reduce telomere length (Figure 3). For instance, chronic inflammation due to CMV, HIV, herpes, and hepatitis B and C infections shortens telomeres’ length [101]. One of the important functions of lengthier telomeres is the expression of long non-coding RNAs regulating the expression of NF-kb, MYC, VEGF, and DNMTs involved in inflammation [102]. Telomere length also affects the expression of many other genes by looping and other mechanisms [102]. 

While telomerase enzymes protect against telomere shortening, and cells with more telomerases have longer telomeres, the short-term use of cooked *brassica* leafy vegetables could increase telomerase activity in CD8+ lymphocytes in humans [103]. Moreover, there is evidence that the chemical compounds of *Astragalus membranaceus* activate telomerase, inhibit senescence, and have neuroprotective effects [104]. *Eucalyptus camaldulensis* bark extract increases telomerase expression, exhibiting anti-aging, anti-apoptosis, and anti-senescence effects as well [105]. In an in silico study, acacetin-7-O-β-D-glucoside, a compound of *Thunbergia erecta*, was also seen to increase telomerase activity in addition to inhibiting acetylcholinesterase (AChE), suggestive of its possible use for the treatment of AD [106]. However, it is important to note that, similar to stem cells, telomerase activity is higher in almost 90% of human cancers [107]. Hence, there have been concerns that using phytochemicals or drugs to increase telomerase activity or expression might have double-edged sword effects. Meanwhile, recent studies found that the non-conical functions of the telomerase TERT subunit include the reduction in mitochondrial oxidative stress, DNA damage, and apoptosis as well as neuronal degradation induced by toxic proteins such as α-synuclein, Aβ, and pathological tau, along with the activation of neurotrophic factors and autophagy, all contributing against age-related neurodegenerative diseases [108]. In this line, recent animal and in vitro studies support that a Jing Si herbal drink increases autophagic clearance in neurons and maintains stem cell homeostasis while suppressing cancer cell growth and migration [109].

Regarding the epigenetic alterations of telomeres and neurodegenerative diseases, a recent study has shown that POT1, encoding one of the telomere capping proteins (which binds to TTAGGG sequences) exhibits DNA methylation alteration in blood cells of patients with dementia as well as those who develop dementia in older ages [110]. Nevertheless, a recent animal study indicated that physical exercise can increase POT1 expression in blood cells [111]. Other lines of evidence indicated that telomeres maintenance is supported by the Mediterranean diet as well [112].

### 3.4. Gut Microbiome, the Dysfunction of Brain Microglia and Astrocytes in Brain Aging, and Phytochemical Effects 

Similar to the brain, the enteric peripheral nervous system also contains glial cells, which are reactive to the gut microbiota composition, infection, and inflammation [113]. While the dysfunction of glial cells has been shown in neurodegenerative diseases (described above), an altered gut microbiome has been repeatedly reported in AD and PD [114,115,116,117]. Furthermore, it has been shown that fecal transplantation from old mice to young mice impacts their performance in spatial learning and memory tests. This is associated with the altered expression of hippocampal proteins involved in neurotransmission and synaptic plasticity along with the aging-like phenotypes of the recipient mice microglia in the hippocampus fimbria [118]. 

Regarding the mechanisms that are involved in the functional status of different tissues/cells as the result of microbiome alteration, it has been shown that the gut microbiota community affects blood inflammatory cytokines. For example, one study from Finland reported a reduced abundance of *Prevotellaceae* (almost 75%) in PD [119] and another study from Taiwan reported a less abundance of *Prevotella* (a genera of *Prevotellaceae*) but more abundance of *Verrucomicrobia*, *Mucispirillum*, *Porphyromonas*, *Lactobacillus*, and *Parabacteroides* in the feces of patients with PD [116]. These microbial alterations were correlated with the plasma levels of IFN-γ and TNF-α in the patients [116]. In particular, the fecal abundances of *Bacteroides* and *Verrucomicrobia* were highly correlated with TNF-α and IFN-γ, respectively [116]. Interestingly, another study reported that a larger number of microbial 16S rRNAs are present in the serum of patients with Parkinson’s disease compared to control subjects [120]. This indicates that specific gut bacteria may cross the intestinal wall (likely due to a leaky gut) and induce inflammatory reactions leading to increased serum levels of inflammatory cytokines.

It is important to note that, as different communities live in diverse environmental milieu with different nutritional habits and genetics, it is conceivable that various communities exhibit different levels of vulnerability to the microbial compositions associated with specific diseases, yet there might be some similarities, as shown in Table 3. For example, regarding PD, an increased abundance of *Lactobacillus* was reported in the fecal samples of patients from Japan, Russia, and Taiwan but not in Finland and the USA. However, a decreased abundance of *Prevotellaceae* has been reported in Finland, Russia, and Taiwan. Regarding AD, a study of Chinese patients reported increased abundances of *Bifidobacterium*, *Sphingomonas*, *Lactobacillus*, and *Blautia* and decreased abundances of *Odoribacter*, *Anaerobacterium*, and *Papillibacter* versus the control subjects [121]. However, in Turkey, higher abundances of *Bacteroides* and *Prevotella* were reported in AD patients [122]. In Americans, the increased abundance of gut *Bacteroidetes* (similar to Turkey), *Blautia* (similar to the Chinese), and *Alistipes* but decreased abundances of *Bifidobacterium* (versus the Chinese) and *Firmicutes* were attributed to AD pathogenesis [115]. Likewise, another study in Americans found an increased proportion of *Bacteroides*, along with *Alistipes*, *Odoribacter*, and *Barnesiella*, and a decreased proportion of *Lachnoclostridium* in elderly AD patients. Meanwhile, this bacterial profile represented a higher abundance of the taxa involved in proinflammatory states and a lower proportion of bacteria synthesizing butyrate, a short-chain fatty acid (largely produced from indigestible fibers) with well-known epigenetic effects [114]. Several other lines of evidence also indicate that a dynamic interplay between food content and gut microbiota continuously affects epigenetic mechanisms. In fact, the interaction between food and the gut microbiome is essential for producing the necessary factors (e.g., folic acid, vitamin B12, choline, and SCFA) that contribute to epigenetic modifications [123]. 

Other studies have shown that microglia are key elements of the “gut–brain axis” in transmitting the impacts of gut microbiota alterations into the brain tissue. For instance, a recent study reports that “gut microbiota-driven brain Aβ amyloidosis in mice requires microglia” to become established [129]. Conversely, treatment with specific bacterial species such as *Clostridium butyricum* could prevent microglia activation and Aβ deposits and have been associated with a reduction in inflammatory cytokines and an improvement in cognitive functions in a mouse model of AD [130]. 

Altogether, these lines of evidence indicate that abnormal gut microbiota may induce astroglia inflammation as well as inflammation-induced oxidative stress, which may alter the epigenetic landscapes of microglia or astrocytes triggering brain pathologies. On the other hand, emerging evidence supports that probiotics, diet, and phytochemicals modulate the gut microbial composition and could be useful in the prevention or treatment of AD and age-associated neurodegenerative diseases [131,132,133,134]. For example, in a multicenter randomized, double-blind, placebo-controlled study, 12-week probiotic treatment in elder South Korean adults could improve mental flexibility and decrease their stress score, increase BDNF serum levels, and decrease the relative abundances of *Eubacterium*, *Allisonella*, *Clostridiales*, and *Prevotellaceae* in their feces [135]. Furthermore, a recent systemic review concluded that while the Mediterranean diet was linked to a lower risk of AD and PD, the abundance of eight bacterial species associated with AD or PD was modulated by this type of diet [136]. Moreover, an animal study revealed that the Qisheng Wan formula, which comprises seven herbal drugs, adjusted the diversity and composition of gut microbiota, decreased Aβ_1–42_ deposition and NF-κb, TNF-α, and IL-6 expression, and improved cognitive functions in a rat model of AD [137]. Poria cocos, a fungus in the family *Polyporaceae* which has been associated with the improvement in cognitive functions, was also seen to improve gut dysbiosis along with reduction of Aβ formation and increase in Aβ clearance in a mouse model of AD [138]. 

### 3.5. Metabolic Disease, Caloric Restriction, Physical Exercise, and Aging 

As obesity is a well-known factor for AD pathogenesis [139] and overeating results in insulin resistance and mTOR activation [140], caloric restriction is a well-documented remedy to mitigate the aging process by reducing the concentration of glucose, lipids, and amino acids and increasing some metabolites such as NAD+ and AMP which modulate SIRT1, AMPK, mTOR, and IGF1 activities. It has also been associated with the improvement of mitochondrial physiology and hemostasis by influencing the transcription factors FOXO and PGC1a [141]. Among commonly used drugs, metformin, an antidiabetic drug originally derived from the *Galega officinalis* plant, could act on multiple pathways targeting aging and age-related diseases by activating AMPK and SIRT1 upregulation and inhibiting mTOR and ROS as well as NF-kb signaling, among others [142]. In addition to metformin, resveratrol, epicatechin, and the NAD+ precursor nicotinamide riboside (a vitamin B3 derivative) have been seen to improve mitochondria functions through similar mechanisms [143,144,145]. Like metformin, physical exercise also increases AMPK which upregulates IL15 in muscle while both AMPK and IL15 activities are decreased by aging [146]. Physical exercise further mitigates the aging process by attenuating age-related chronic and sterile inflammation and immunosenescence that affect mitochondrial functions, while it is intensified by mitochondria damage [147]. In light of these findings, it is intriguing to note that, like physical exercise, certain phytochemicals such as curcumin could reverse the expression alterations of hundreds of genes affected in AD [148].

### 3.6. Chromosome X Inactivation and Neurodegeneration 

Since AD is more common in women [149], there have been efforts to uncover the underlying mechanisms of this difference versus men. In this line, XIST, a long non-coding RNA that regulates the inactivation of one X chromosome in female cells, was found to be an important player involved in female AD pathogenesis. XIST expression is increased in the aged female brain and the entorhinal cortex of female patients with AD [150]. Single-nuclei RNA sequencing revealed that XIST expression is elevated with age in the hypothalamic neurons of female mice and that it can be considered a powerful predictor of neuronal aging [151]. It has also been shown that XIST induces Aβ accumulation and neuroinflammation in the AD mouse model [152]. Although there is no study to confirm that phytochemicals may suppress XIST expression, one study reports that the expression of XIST correlates directly with the blood glucose levels in gestational diabetes mellitus [153]. This suggests that caloric restriction using fiber-rich foods or the Mediterranean diet and efficient nutritional management of diabetes (which is associated with a higher risk of AD) may be promising approaches in suppressing XIST expression. However, more research to study the potential effects of specific phytochemicals on XIST expression in females is warranted. 

### 3.7. Vascular System and Neurodegeneration 

Dysfunction of the vascular system and endothelial cells is linked to neuronal degeneration as well. For example, it has been shown that in aged or irradiated mice, the production of TGFB1 is increased in the endothelial cells, causing neuronal stem/progenitor cell apoptosis which can be inhibited by selective TGFB signaling inhibitors [154]. Dyslipidemia can also lead to the senescence of endothelial cells which, in addition to atherosclerosis, affects their integrity and permeability [155], thus impacting the functionality of the BBB and influencing brain functions. Furthermore, complement C3, which is increased in the hippocampal astrocytes by aging, affects C3aR1 on the endothelial cell surface, causing inflammation and vessel permeability [156]. As mentioned before, while withaferin A and active fractions of golden-flowered tea inhibit TGFB1 expression [48,49], the active compounds of black chokeberry (*Aronia melanocapa* L.) decrease the expression of inflammatory factors, including C3 receptors, in neuronal cells [45].

## 4. Conclusions

Several factors and genes are linked to aging and aging-associated neurodegeneration and there are more than two dozen known phytochemicals that may modulate the affected molecular and cellular targets. Although the main focus of this review was on the potential effects of phytochemicals and diet in more common brain neurogenerative diseases (e.g., AD and PD), there is also limited evidence that phytochemicals might be effective in other neurogenerative diseases such as Huntington’s disease and those diseases involving oligodendrocyte dysfunction [157,158,159]. However, the experimental evidence from preclinical studies remains limited to defining the efficacy and safety of those phytochemicals, alone and in combination, in preventing the development and progression of aging and associated neurodegenerative diseases. Furthermore, there have been many compounds that, although effective in vitro, could not cross the BBB in preclinical studies or were rapidly metabolized by the liver or the gut microbiome and are thus ineffective in vivo. Consequently, new approaches are necessary to modify these compounds to increase their functional half-life and enhance their ability to penetrate the BBB. Some of these compounds even had harmful side effects in other tissues, limiting their use in humans. Therefore, in addition to an examination of their safety profiles and potency for crossing the BBB, one of the future research priorities should be to define the synergistic combination regimens of phytochemicals as novel nutritional strategies for the effective and safe management of aging and aging-associated neurodegenerative disorders. Next, their industrial purification/synthesis and determination of their appropriate dosages may help to produce new drugs for neurodegenerative diseases. Additionally, considering the close interaction between nutrition and the gut microbiome composition and the diverse gut microbial alterations associated with PD and AD in different communities/cultures and geographical locations with diverse nutritional habits, community-specific interventions using probiotics and modified diets may further help to prevent or delay the progression of neurogenerative diseases.

## Figures and Tables

**Table 1:** Phytochemicals that modulate neuroinflammation and brain aging-related genes in neurodegenerative diseases.

Phytochemicals or Nutrients	In Vitro and/or Animal Models	Effects	Mechanisms of Action	References
Anthocyanins (bilberry)	APP/PSEN1 transgenic mouse model of AD	- Decrease hippocampal neuroinflammation - Improve cognitive functions - Increase microglia Aβ phagocytosis	- Upregulate TREM2/TYROBP/CD33 signaling pathway	[22]
Cyanidin-3-O-glucoside (fruits and vegetables)	Mouse model of AD	- Reduces ROS and neuroinflammation - Increases microglial Aβ42 phagocytosis	- Upregulates TREM2 expression	[23]
Fucoidan (Atlantic brown algae)	*D. melanogaster* and mice	- Increases lifespan - Alleviates LPS-induced cognitive impairment in mice	- Increases SIRT6 expression	[39,40,42]
Bioactive compounds in black chokeberry (*Aronia melanocapa* L.)	Neuronal cells and mice brain	- Decrease cell death and Aβ-induced neuronal cell death - Improve the benefits of exercise on neurodegeneration	- Decrease the expression of ITGB2/C3R and several other inflammatory factors	[45]
Curcumin	In vitro and in vivo	- Inhibits Aβ plaque formation - Decreases hyperphosphorylated tau and increases its elimination - Decreases microglial activity	- Reduces acetylcholinesterase and ROS	[76]
Curcumin	Animal model of spinal cord injury	- Suppresses inflammation and reactive gliosis	- Decreases TGF-β1 and TGF-β2, TNF-α, IL-1β, and NF-κb	[51]
Galangin	Rat brain	- Inhibits astrocytic activation and neuroinflammation - Improves cognitive– behavioral functions	- Suppresses the HMGB1/TLR4 bond - Reduces inflammatory cytokines - Increases BDNF	[58]
Astin C (a cyclopeptide from the *Aster tataricus* plant)	Mice	- Decreases the innate inflammatory responses triggered by cytosolic DNA	- Inhibits cGAS-STING signaling	[68]
Apigenin (parsley and celery)	iPSC-derived neurons from patients with AD	- Neuroprotective via decreasing inflammation and ROS	- Downregulates cytokines and nitric oxide release and Ca(2+) signals	[73]
Resveratrol (grapes and berries)	Yeast and flies	- Inhibits inflammation - Delays the aging process	- Inhibits the expression of NFkB and iNOS	[74]
EGCG (tea polyphenol)	APP/PS1 mouse model of AD	- Reduces Aβ (1–40), APP, and neuronal apoptosis - Improves cognition	- Activates TrkA signaling (the receptor for BDNF)	[75]
Acacetin (*Robinia pseudoacacia* plant)	MPTP-induced mouse model of PD and LPS-induced mouse model of neuroinflammation	- Has anti-inflammatory activity - Protects dopamine neurons against MPTP neurotoxicity - Suppresses microglial activation and neuronal cell death	- Inhibits the production of inflammatory factors (nitric oxide, prostaglandin E2, TNF-α, and IL-1β) - Inhibits NFkB activation	[77,78]
Phytic acid (plants and seeds) and aspirin	Neuronal cells and aged mice brain	- Improve cognition in aged mice - Inhibit senescence - Protect against APP-C-terminal fragment-induced cytotoxicity	- Upregulate SIRT1 expression	[43,44]

**Table 3:** Similarities and differences of microbiota alterations in Parkinson’s disease (PD) and Alzheimer’s disease (AD) in different countries.

Disease	Country	Increased	Decreased	Reference
PD	Finland	-	*Prevotellaceae* *	[119]
PD	USA		*Blautia*, *Coprococcus*, and *Roseburia*	[124]
PD	Japan	*Lactobacillus* *	*Clostridium coccoides* * and *Bacteroides fragilis* *	[125]
PD	Russia	*Lactobacillus* *, *Bifidobacterium*, and *Papillibacter cinnamivorans* among others	*Dorea*, *Bacteroides*, *Prevotella* *, *Coprococcus eutactus*, and *Ruminococcus callidus*, among others	[126]
PD	China	*Alistipes*, *Paraprevotella*, *Klebesiella*, *Sphingomonas*, *Acinetobacter*, *Aquabacterium*, *Desulfovibrio*, *Clostridium IV*, *Lachnospiracea incertae sedis*, *Butyricicoccus*, *Clostridium XVIII*, and *Nitrososphaera*	*Lactobacillus* ^¥^ and *Sediminibacterium*	[127]
PD	Taiwan	*Verrucomicrobia*, *Mucispirillum*, *Porphyromonas*, *Lactobacillus* *, and *Parabacteroides*	*Prevotella* * (a genera of *Prevotellaceae*) *	[116]
PD	Italy		*Lachnospiraceae*	[128]
AD	USA	*Bacteroidetes* *, *Blautia*^®^, and *Alistipes*^©^	*Bifidobacterium* ^¥^, *Firmicutes*, and *Actinobacteria*	[115]
AD	USA	*Bacteroides* *, *Alistipes*^©^, *Odoribacter* ^¥^, and *Barnesiella*	*Lachnoclostridium*, *Butyrivibrio*, and *Eubacterium*	[114]
AD	China	*Bifidobacterium* ^¥^, *Sphingomonas*, *Lactobacillus*, and *Blautia*^®^	*Odoribacter* ^¥^, *Anaerobacterium*, and *Papillibacter*	[121]
AD	Turkey	*Bacteroides* * and *Prevotella*		[122]

* Indicates similarity in PD or AD; ^®^ and ^©^ indicates similarity in AD; and ^¥^ indicates differences in AD.

## Data Availability

Not applicable.

## Supplementary Materials

The following supporting information can be downloaded at: https://www.mdpi.com/2072-6643/15/15/3456/s1, Table S1: Number of articles reviewed in PubMed for this work using each primary key words plus one of the secondary key words.
