# Supplementary material for: Underlying Mechanisms of Brain Aging and Neurodegenerative Diseases as Potential Targets for Preventive or Therapeutic Strategies Using Phytochemicals
Source: Nutrients. 2023 Aug 4;15(15):3456. doi: 10.3390/nu15153456 (PMC10473240; doi:10.3390/nu15153456)
Supplement: Supplementary file 1 [file nutrients-15-03456-s001.zip › nutrients-2510539-supplementary.pdf]

Supplementary Table S1. Number of articles reviewed in PubMed for this work using each primary key words plus one of the secondary key words.

	Secondary key words			
Primary key word(s)	Gene Expression	Epigenetic	Microbiome	Total articles
Brain Aging	458	70	40	568
Neurodegeneration	520	56	31	607
Alzheimer's disease	660	92	71	823
Parkinson's disease	370	42	58	470
Total articles	2008	260	200	**2468**
